# The Expression of Toll-like receptors in eutopic and ectopic endometrium and its implication in the inflammatory pathogenesis of adenomyosis

**DOI:** 10.1038/s41598-017-07859-5

**Published:** 2017-08-04

**Authors:** Caixia Jiang, Chao Liu, Jing Guo, Li Chen, Ning Luo, Xiaoyan Qu, Weihong Yang, Qing Ren, Zhongping Cheng

**Affiliations:** 1 Department of Gynecology and Obstetrics, Yangpu Hospital, Tongji University School of Medicine, 450 Teng Yue Road, Shanghai, 200090 China; 2 Institute of Gynecological Minimal Invasive Medicine, Tongji university School of Medicine, 450 Teng Yue Road, Shanghai, 200090 China; 3 Department of Gynecology and Obstetrics, Shanghai Ninth People’s Hospital, Shanghai JiaoTong University School of Medicine, 280 Mo He Road, Shanghai, 201999 China

## Abstract

In this study, we investigated the expression profiles of Toll-like receptors(TLRs) in eutopic endometrium(EU) and ectopic endometrium(EC) and its implication in the inflammatory pathogenesis of adenomyosis. Thirty adenomyosis patients who underwent laparoscopy were recruited in this study. We tested the mRNA and protein expression of TLRs, and the mRNA expression of IL-6 and IL-8 in EU and EC of adenomyosis patients, and control endometrium without adenomyosis(CE). We found that the mRNA expression of IL-6 and IL-8 in EU was significantly higher than that in CE, and was the highest in EC (*P* < 0.01). The mRNA and protein expression of TLRs were higher in EU, with the expression of TLR1-6, 8 and 9 being significantly higher in EU than in CE, and were the highest in EC (except TLR6) (*P* < 0.05 or *P* < 0.01). Pearson correlation analysis showed that the expression of TLR1, 2, 4, 5 and 9 in EU and EC was positively correlated with that of IL-6 and IL-8 (*P* < 0.00139). This study suggested that adenomyosis was a state of inflammatory pathology. High expression of TLRs in EU and EC were positively correlated with IL-6 and IL-8, which may be involved in the inflammatory pathogenesis of adenomyosis.

## Introduction

As a special form of endometriosis, adenomyosis is characterized by the presence of heterotopic endometrial glands and stroma within the myometrium^1^. Emerging evidence suggests that adenomyosis is a type of chronic inflammatory disease^2, 3^. Adenomyosis tissues are rich in inflammatory immune cells including macrophages, dendritic cells (DCs) and neutrophils, and inflammatory cytokines including IL-6, IL-8, TNF-α and COX-2^4, 5^. The inflammatory pathology plays a major role in the development of adenomyosis and is related to the clinical manifestations such as dysmenorrhea and subfertility^6^. Recent evidence indicates that the inflammatory pathogenesis of this disease may be related to the expression of inflammatory mediators, induction of immune response, and genital tract infections as well^7, 8^.

Toll-like receptors (TLRs) are essential components of the innate immune system that protect the host against bacterial and viral infection^9^. It is well known that TLRs are type 1 transmembrane proteins with an N-terminal extracellular domain characterized by leucine-rich repeats (LRRs), a singular transmembrane helix, and a C-terminal cytoplasmic Toll/IL-1 receptor (TIR) domain. LRRs shape like a horseshoe structure, and their ligand-binding with TLRs forms heterodimers or homodimers, which can initiate signaling cascade^10^. TLR-mediated activation of NF-κB is considered an essential link connecting inflammation to cancer^11^. Numerous studies have demonstrated that TLR-mediated activation of immune response may be associated with endometriosis and adenomyosis. It was reported that the expression of TLR4 in the ectopic endometrium of endometriosis was higher than that in the normal endometrium, and the levels of TLR2 and TLR9 mRNAs were higher in the peritoneal effusions of endometriosis than in the non-endometriosis group^12, 13^.

We previously demonstrated that TLR4 was over-expressed in adenomyosis tissues, and the cell model indicated that stromal cells were activated by TLR4 signaling pathway, which processed the cellular inflammatory proliferation and invasive growth involved in the pathogenesis of adenomyosis^14^. However, the exact inflammatory pathogenesis of TLR-mediated activation of immune response implicated in the development of adenomyosis remains unclear, and no study to date has reported the expression profiles of TLRs in adenomyosis by measuring all TLRs simultaneously. The aim of the present study was to investigate the expression profiles of TLRs in eutopic endometrium (EU) and ectopic endometrium (EC) to see whether they were truly involved in the inflammatory pathogenesis in adenomyosis.

## Materials and Methods

### Ethics statement

The study was approved by the Institutional Review Board of the Yangpu Hospital, Tongji University School of Medicine, Shanghai, China. All biopsy specimens were collected according to the guidelines of the Declaration of Helsinki. Written informed consent was obtained from all subjects participating in the study.

### Participants

From September 10, 2015 to November 12, 2016, 30 patients who underwent laparoscopic surgery due to adenomyosis at the Gynecological Department of Yangpu Hospital, Tongji University School of Medicine were included in this study. The diagnosis was confirmed by histology. Healthy women who underwent intrauterine device (IUD) placement were recruited as control group (n = 30). All patients enrolled in this study were in the proliferative stage. Participants with regular menstrual cycles (28–32 days), were not administered any hormonal therapy including oral contraceptive pills, progestins, gonadotropin-releasing hormone agonist, or levonorgestrel intrauterine system (LNG-IUS) in recent 3 months. Individuals with cancer, cardiovascular diseases, autoimmune diseases, endocrine diseases, metabolic diseases, pelvic inflammatory disease, and other infectious diseases were excluded.

### Tissue collection

EU and EC samples were derived from adenomyosis patients, and endometrium samples without adenomyosis (CE) were derived from healthy controls under strict asepsis. All the fresh tissue specimens were frozen on liquid nitrogen and stored at −80 °C for RNA and protein extraction.

### RNA extraction and real-time PCR

Total RNA was extracted from the frozen tissue samples using Trizol reagent (Invitrogen, Waltham, MA, USA). Complementary DNA was synthesized by reverse transcriptase (Fermentas, Waltham, MA, USA) at 42 °C for 1 h and 75 °C for 5 min. Primers specific for TLR1-9, IL-6, and IL-8 are shown in Table 1. Real-time PCR was performed with SYBR-Green Master Mix on an ABI7300 platform and related software (Thermo Fisher Scientific, Waltham, MA, USA). β-actin was used as an internal control. The PCR cycling conditions were as follows: 94 °C for 7 min, followed by 40 cycles of 15 s at 94 °C and 60 °C for 45 s. ΔCt was defined as the difference in the cycle threshold between the target gene and internal control, and ΔΔCt was defined as the difference between the ΔCt values of the test sample and control. The relative expression of the target genes was calculated as 2^−ΔΔCt^.

**Table 1:** Primers for real-time RT-PCR.

Name	Sequences (5′-3′)	Annealing temperature (°C)	Product size (bp)
TLR1	F: AGGGTCAGCTGGACTTCAGA	60	215
	R: AAAATCCAAATGCAGGAACG		
TLR2	F: ATTGTGCCCATTGCTCTTTC	60	311
	R: CTGCCCTTGCAGATACCATT		
TLR3	F: TTGCCTTGTATCTACTTTTGGGG	61	157
	R: TCAACACTGTTATGTTTGTGGGT		
TLR4	F: CCGCTTTCACTTCCTCTCAC	60	167
	R: CATCCTGGCATCATCCTCAC		
TLR5	F: GAGCCCCTACAAGGGAAAAC	61	184
	R: TGCTGATGGCATTGCTAAAG		
TLR6	F: GGATAGCCACTGCAACATCA	60	175
	R: CAGCGGTAGGTCTTTTGGAA		
TLR7	F: AATGTCACAGCCGTCCCTAC	61	185
	R: TTATTTTTACACGGCGCACA		
TLR8	F: TCCTTCAGTCGTCAATGCTG	61	167
	R: CGTTTGGGGAACTTCCTGTA		
TLR9	F: AAAGAGGAAGGGGTGAAGGA	60	211
	R: GACAGCAGCTACAGGGAAGG		
IL-6	F: AGCCACTCACCTCTTCAGAAC	60	118
	R: GCCTCTTTGCTGCTTTCACAC		
IL-8	F: CAAGAGCCAGGAAGAAAC	60	227
	R: TGGTCCACTCTCAATCAC		
β-actin	F: CAAGATCATTGCTCCTCCTG	60	90
	R: ATCCACATCTGCTGGAAGG		

Note: RT-PCR, real time-polymerase chain reaction; TLR, Toll-like receptor; IL, interleukins.

### Western blot analysis

Protein was extracted with radio-immunoprecipitation assay(RIPA) buffer containing a protease inhibitor cocktail and centrifuged at 12,000 × g for 15 min at 4 °C. The supernatant protein was quantified by bicinchoninic acid assay (BCA, Thermo Fisher Scientific, Rockford, USA) and stored at −80 °C. Total lysates were resolved in SDS-PAGE. Proteins were blotted onto a nitrocellulose membrane and incubated at 4 °C overnight with one of the following primary antibodies: anti-TLR1 (1:500, ab180798), anti-TLR2 (1:100, ab16894), anti-TLR3 (1:1000, ab62566), anti-TLR4 (1:500, ab13556), anti-TLR5 (1:1000, ab62460), anti-TLR7 (1:1000, ab45371), anti-TLR8 (1:1000, ab53630), anti-TLR9 (1:800, ab12121) (Abcam, Cambridge, MA, USA), anti-TLR6 (1:1000, av31755, Sigma-Aldrich Corporation, St. Louis, MO, USA), GAPDH (1:2000, 5174, CST, Danvers, MA, USA), diluted in TBS containing 1% skimmed milk. The blots were then washed with TBS, incubated with the corresponding secondary antibodies (HRP-labeled Goat Anti-Rabbit IgG (H+L), a0181; HRP-labeled Donkey Anti- Goat IgG (H+L), a0181; HRP-labeled Goat Anti-Mouse IgG (H+L), a0216) (Beyotime Institute of Biotechnology, Haimen, China) at room temperature for 1 h. The optical density method of protein signal strength was performed by using the enhanced chemiluminescence western blotting system (BioRad, Richmond, CA), and GAPDH served as an internal standard to determine the protein level.

### Statistical analysis

All statistical analyses were performed with SPSS version 16.0 for Windows (SPSS Inc.,Chicago, IL, USA). The normality test of the measurement data is conducted by using the kurtosis and skewness coefficients and the Shapiro-Wilks test. Normally distributed data were presented as means ± standard deviations, and intragroup differences were investigated using analysis of variance (ANOVA) or Student *t* test when necessary. Non-normally distributed data were presented as median (quartiles), and intragroup differences were made using Kruskal-Wallis or Mann-Whitney test when necessary. Categorical variables were expressed as the number of cases and percentages (%). Correlations of variables for each group were determined using Pearson correlation coefficient (r) or Spearman rank correlation coefficient (r_s_) when necessary, and Bonforoni correction was used to determine the *P* value for significance(*P* < 0.00139). A value of *P* ≤ 0.05 (2-sided test) was considered statistically significant. Statistics diagrams were performed with GraphPad Prism 5.

## Results

### General data of patients in the adenomyosis and non-adenomyosis groups

The 30 adenomyosis patients included 3 nulliparas and 27 multiparas who ranged in age from 32 to 52 years with a mean of 42.48 ± 5.28 years and a mean body mass index (BMI) of 23.28 ± 2.71 (18.73–28.89) kg/m^2^. The 30 non-adenomyosis subjects were all multiparas who ranged in age from 25 to 47 years with a mean of 38.69 ± 9.12 years and a mean BMI of 22.24 ± 3.31 (18.41–28.31) kg/m^2^. There were no statistically significant differences in age, BMI, fertility, or history of prior surgery between the groups (*P* > 0.05 each).

### Clinical characteristics of the adenomyosis group

The mean history of the patients with adenomyosis was 13.54 ± 5.13 (1–240) months. According to the visual analog scale (VAS) system^15^, there were 6 (20.00%) cases of minimal dysmenorrhea, 9 (30.00%) cases of moderate dysmenorrhea, and 15 (50.00%) cases of severe dysmenorrhea. According to the pictorial blood loss assessment chart (PBAC)^15^, there were 17 (56.67%) cases of normal menstrual capacity and 13 (43.33%) cases of menorrhagia. The mean uterine volume of adenomyosis group was 225.16 ± 101.83 (82.33–522.64) cm^3^.

### Expression of IL-6, IL-8 and TLRs mRNAs in CE, EU and EC

**Figure 1:**
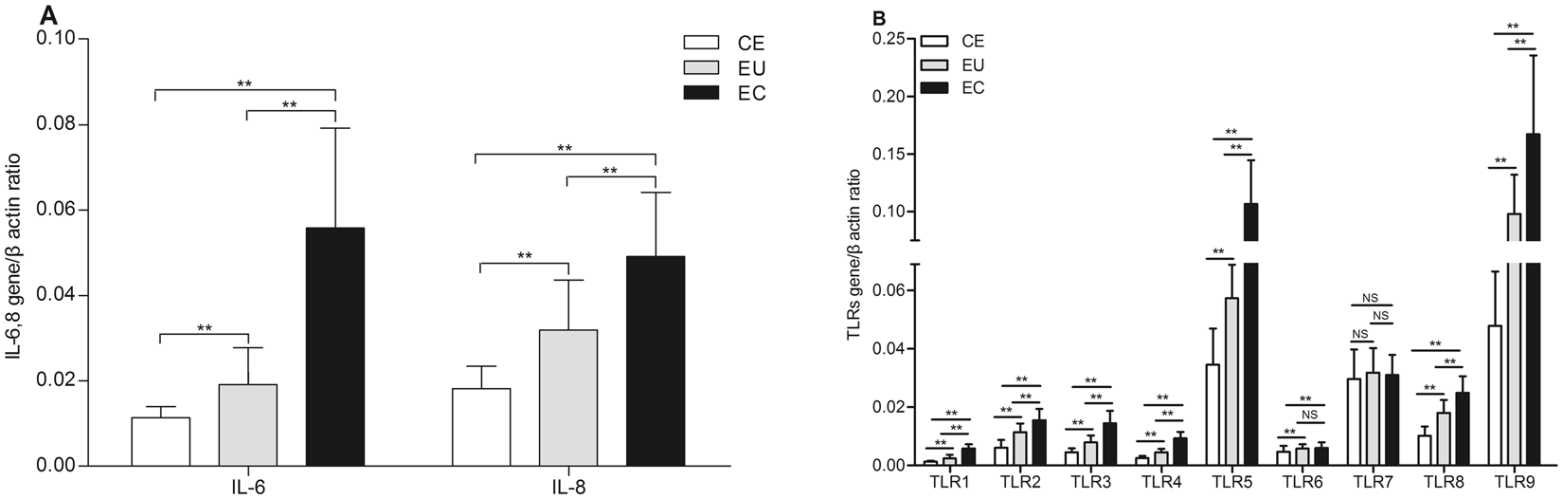
mRNAs expression of IL-6, IL-8 and TLRs in CE, EU and EC. CE: endometrium without adenomyosis; EU: eutopic endometrium with adenomyosis; EC: ectopic endometrium with adenomyosis; NS *P* > 0.05; *P < 0.05; **P < 0.01.

RT-PCR analysis showed that the mRNAs expression of IL-6 and IL-8 in EC and EU was significantly higher than that in CE, and their expression in EC was significantly higher than that in EU (*P* < 0.01). The mRNAs expression of TLR1-6, 8 and 9 in EC and EU was significantly higher than that in CE (*P* < 0.01), and their expression (except TLR6) in EC was significantly higher than that in EU (*P* < 0.01). And there was no significant difference in TLR7 expression between EU *vs*. CE, EC *vs*. CE, EU *vs*. EC (Fig. 1 **)**.

### Expression of TLRs protein in CE, EU and EC

**Figure 2:**
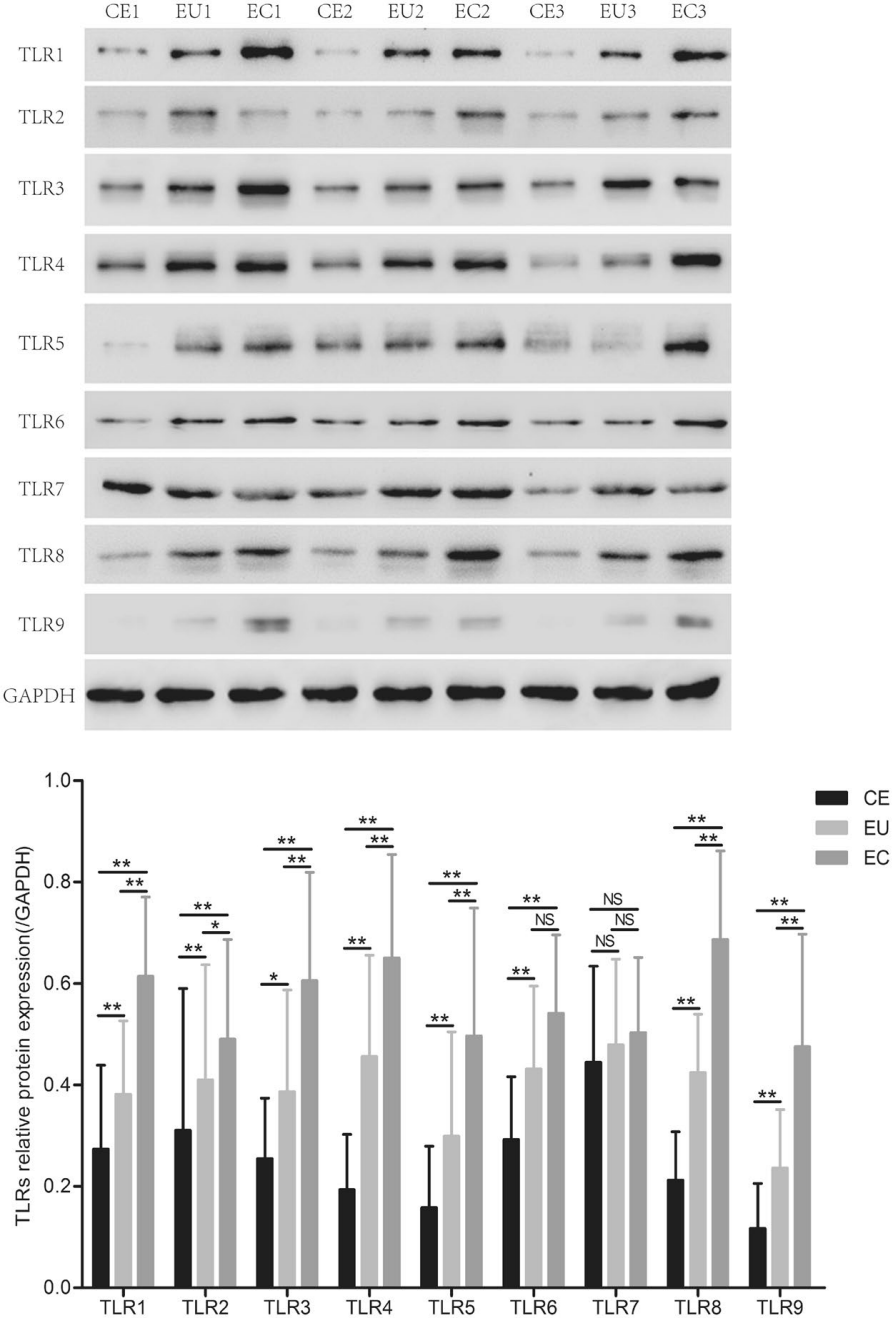
Protein expression of TLRs in CE, EU and EC. CE: endometrium without adenomyosis; EU: eutopic endometrium with adenomyosis; EC: ectopic endometrium with adenomyosis. NS *P* > 0.05; *P < 0.05; **P < 0.01.

Western blot analysis was performed to detect the expression of TLRs in CE, EU and EC. The protein expression of TLR1-6, 8 and 9 in EC and EU was significantly higher than that in CE, and their expression (except TLR6) in EC was significantly higher than that in EU (*P* < 0.05 or *P* < 0.01). And there was no significant difference in TLR7 expression between EU *vs*. CE, EC *vs*. CE, EU *vs*. EC (Fig. 2).

### Correlations between the mRNA expressions of IL-6 and TLRs in adenomyosis

**Figure 3:**
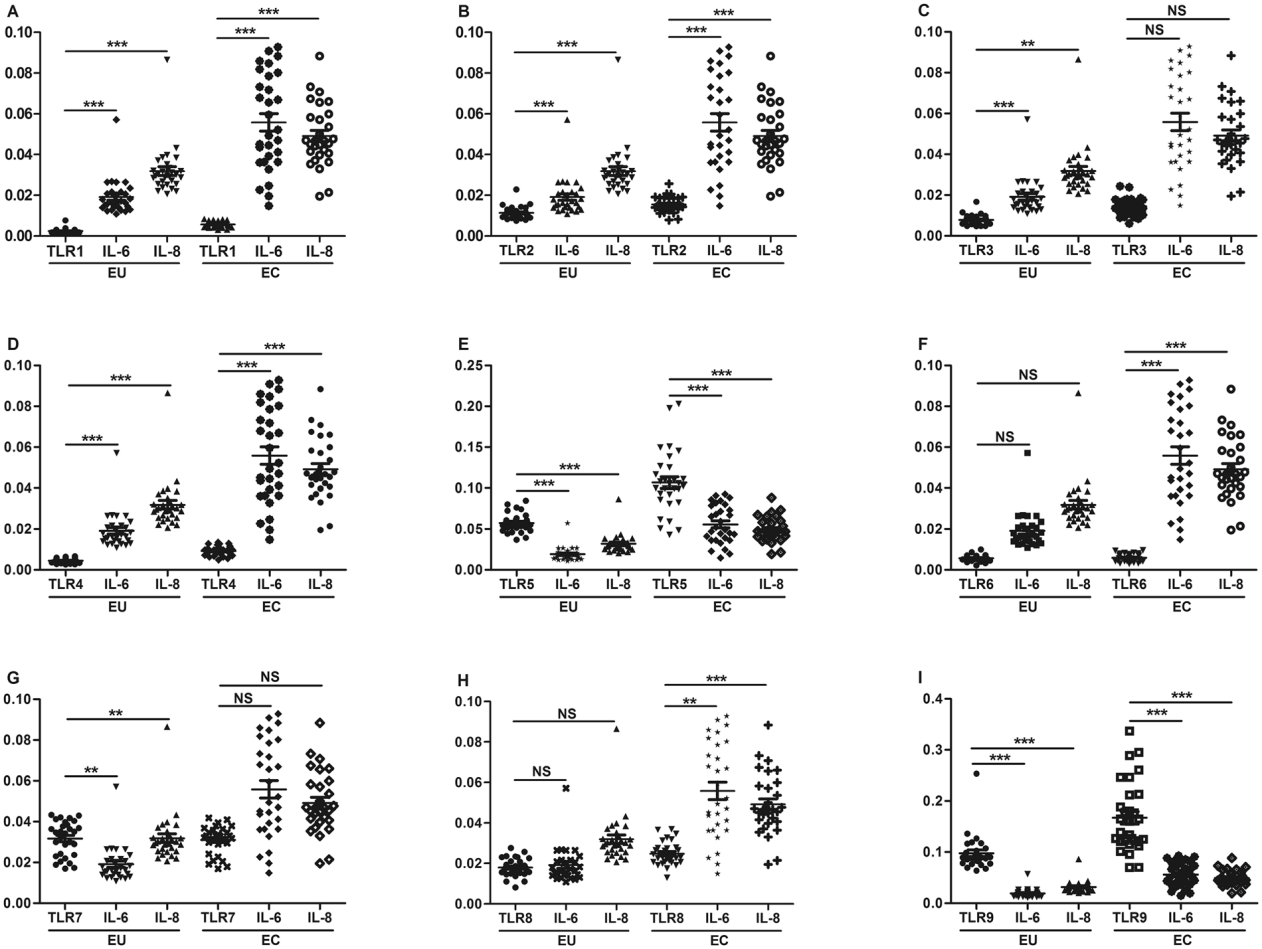
Correlations between the mRNAs expression of IL-6, IL-8 and TLRs in adenomyosis. CE: endometrium without adenomyosis; EU: eutopic endometrium with adenomyosis; EC: ectopic endometrium with adenomyosis. NS *P* > 0.05; *P < 0.05; **P < 0.01; ***P < 0.00139.

There was a significant positive correlation between the mRNA expressions of IL-6 and TLR1-4, 5 and 9 in EU (*P* < 0.00139), and the mRNA expression of IL-6 was positively correlated with that of TLR1, 2, 4, 5, 6 and 9 in EC (*P* < 0.00139), while it showed no significant correlation with the other TLRs. The value of r or r_s_ are presented in Table 2 (Fig. 3).

**Table 2:** Correlations between the mRNAs expression of IL-6 and IL-8 and TLRs in EU and EC.

		TLR1	TLR2	TLR3	TLR4	TLR5	TLR6	TLR7	TLR8	TLR9
EU	IL-6	0.828***	0.777***	0.557***	0.758***	0.881***	NS	0.497**	NS	0.852***
	IL-8	0.844***	0.776***	0.551**	0.625***	0.844***	NS	0.538**	NS	0.820***
EC	IL-6	0.792***	0.770***	NS	0.748***	0.726***	0.611***	NS	0.535**	0.666***
	IL-8	0.818***	0.805***	NS	0.700***	0.749***	0.677***	NS	0.723***	0.602***

Note: EU: eutopic endometrium with adenomyosis; EC: ectopic endometrium with adenomyosis. NS *P* > 0.05; *P < 0.05; **P < 0.01; ***P < 0.00139.

### Correlations between the mRNAs expression of IL-8 and TLRs in adenomyosis

Data analysis revealed that there was a significant positive correlation between the mRNA expression of IL-8 and TLR1, 2, 4, 5 and 9 in EU (*P* < 0.00139), and the mRNA expression of IL-8 was positively correlated with that of TLR1, 2, 4, 5, 6, 8 and 9 in EC (*P* < 0.00139), while it showed no significant correlation with the other TLRs. The value of r or r_s_ are shown in Table 2 (Fig. 3).

## Discussion

Adenomyosis is a benign invasion of the endometrium into the myometrium that is closely related to endometriosis. As a type of chronic inflammatory disease, the inflammatory pathogenesis of adenomyosis involved in abnormal immune responses and genital tract infections have attracted increasing attention^10, 16^. As indispensable members of pattern recognition receptors (PRRs), TLRs play a major role in endometrial defense against microorganisms. TLRs have also been described as transmembrane proteins that recognize specific pathogen-associated molecular patterns (PAMPs) found in viruses and other invading pathogens. In addition to PAMPs, TLRs recognize damage-associated molecular patterns (DAMP), which are proteins or nucleic acids released during necrosis^17^. PAMPs and endogenous DAMPs bind to TLRs and activate the downstream signaling pathways leading to activation of inflammatory pathways in the endometrium, where they may play a vital role in the inflammatory pathogenesis of adenomyosis.

Recent evidence has revealed that the adenomyosis tissues are infiltrated with inflammatory immune cells and various inflammatory cytokines^4, 5^. Among others, IL-6 and IL-8 have been studied extensively and the most commonly used to demonstrate the inflammatory pathological state of adenomyosis^18^. Luo *et al*.^12^ found that the expression of TLR4 in EU was higher than that in the normal endometrium, and was the highest in EC tissue. TLR4 activation stimulated IL-8 secretion, thus enhancing the invasion and proliferation of endometrial stromal cells through the FAK signal pathway, and these effects could be abolished by an anti-CXCL8 neutralizing antibody or by a FAK inhibitor. It was found in this study that the mRNA expression of IL-6 and IL-8 in EU was significantly higher than that in CE, and was the highest in EC (*P* < 0.01), which is consistent with previous studies, indicating that adenomyosis is an inflammatory pathological state. Furthermore, we tested the mRNA and protein expressions of TLRs in EC, EU and CE and found that the expression of TLRs were higher in EU, with the expression of TLR1-6, 8 and 9 in EU were significantly higher than that in CE, and were the highest in EC (except TLR6). In addition, we analyzed the correlation between IL-6 and IL-8 and TLRs in adenomyosis patients and found that TLRs were positively correlated with corresponding IL-6 and IL-8 in EU and EC, of which TLR1, 2, 4, 5 and 9 were significantly positively correlated with IL-6 and IL-8. We therefore conclude that amplification of TLRs in the inflammatory immune signaling system may result in an inflammatory pathological state of adenomyosis. TLRs can regulate cell proliferation and survival, and create a tumor microenvironment that can facilitate tumor growth by expanding immune cells and integrating inflammatory responses and tissue repair processes^11^. Additionally, we previously found that LPS/TLR4-mediated stromal cells of adenomyosis acquired an invasive phenotype^14^. From the results herein, these same roles in tumor microenvironment further demonstrated that TLRs were involved in the inflammatory pathogenesis of adenomyosis and may be mediated via NK-κB signaling pathway.

The exact pathogenesis of adenomyosis has not yet been fully elucidated. In the recent years, some few domestic studies proposed a novel concept of ‘determinant of uterine eutopic endometrium’ for endometriosis. The author suggested that endometriosis onset or not depends on the biological characteristics of eutopic endometrium, and retrograde menstruation was just a bridge to achieve this process from potential contribution to pathogenesis^19^. In this study, the mRNA expression of IL-6 and IL-8 in both EC and EU was significantly higher than that in CE, indicating that EU was also in an inflammatory state. We further tested the mRNA and protein expressions of TLRs and found that TLRs in both EC and EU were significantly higher than those in CE, and the trend of the relative expression of TLRs in EU was consistent with that in EC, suggesting that the biological characteristics of EU were similar to those of EC, and different from those of CE; the abnormal immune response of EU led to EU activation, which is consistent with the molecular mechanism of this hypothesis. The relationship between the expression of IL-6 and IL-8 and TLRs in EU and the pathogenesis of adenomyosis require more future research.

In addition, patients were divided into several subgroups according to the clinical characteristics in this study. After statistical analysis confirmed that: The gene expression of TLRS (TLRS = TLR1 + TLR2 + TLR3 + TLR4 + TLR5 + TLR6 + TLR7 + TLR8 + TLR9) in severe and moderate dysmenorrhea group was significantly higher than that in minimal dysmenorrhea group in EU and EC (*P* < 0.05 or *P* < 0.01). And the gene expression of TLRS in menorrhagia group were significantly higher than that in normal group in EU and EC (*P* < 0.05 or *P* < 0.01). The correlations between the expression of TLRs and the disease severity suggest that rises in dysmenorrhea and menstrual capacity paralleled increase in the expression of TLRs.

Based on the results obtained from our previous and present studies and literature review, we speculate the following possible pathogenesis of adenomyosis: TLR4-dependent signaling activated by endogenous PAMPs and DAMPs promotes the secretion of different proinflammatory cytokines and chemokines, stimulates endometrial cell proliferation, recruits and activates immune cells (macrophages, DCs, NK cells), triggers local inflammatory response, and activates the fabrication and maintenance of an inflammatory microenvironment by the specific immune system, which further induce stromal cell proliferation and invasion, promote the development and progression of the local inflammatory pathology of adenomyosis, and eventually lead to the development of adenomyosis.

Despite our important findings, there are some limitations in this study. First, we did not detect the peritoneal fluid and serous expression of IL-6, IL-8 and TLRs. In addition, the menstrual dates used in this study were obtained only by review of the medical records without histologic confirmation. Therefore, further studies with larger sample sizes are needed to avoid these limitations and verify the findings of the present study.

In summary, adenomyosis is a common and intractable gynaecological disease mostly affecting women of reproductive age. In this study, the expression of IL-6, IL-8 and TLRs showed an increased tendency in CE, EU and EC, and the expression of TLRs in EU and EC was positively correlated with the expression of IL-6 and IL-8. These findings may provide a theory basis for further revealing the development and progression of the inflammatory pathology, and the underlying immune inflammatory pathogenesis of adenomyosis. Future studies in cell or animal models are required to address the role of TLRs signaling pathways in the inflammatory pathogenesis and the molecular mechanisms of adenomyosis.

### Data availability

All data is available within manuscript.

## Electronic supplementary material

supplementary information
