# Supplementary material for: The Expression of Toll-like receptors in eutopic and ectopic endometrium and its implication in the inflammatory pathogenesis of adenomyosis
Source: Sci Rep. 2017 Aug 4;7:7365. doi: 10.1038/s41598-017-07859-5 (PMC5544718; doi:10.1038/s41598-017-07859-5)

**Title page**

**Title**

**The Expression of Toll-like receptors in eutopic and ectopic endometrium and its implication in the inflammatory pathogenesis of adenomyosis**

**Authors list**

Caixia Jiang^1^, Chao Liu^1^, Jing Guo^1^, Li Chen^1^, Ning Luo^1^, Xiaoyan Qu^1^, Weihong Yang^1^, Qing Ren^3^, Zhongping Cheng^1,2^*

1.Department of Gynecology and Obstetrics, Yangpu Hospital, Tongji University School of Medicine, 450 Teng Yue Road, Shanghai 200090, China

2.Institute of Gynecological Minimal Invasive Medicine, Tongji university School of Medicine, 450 Teng Yue Road, Shanghai 200090, China

3.Department of Gynecology and Obstetrics, Shanghai Ninth People’s Hospital, Shanghai JiaoTong University School of Medicine, 280 Mo He Road, Shanghai 201999, China

Author contact respectively: [m13681983326@163.com](mailto:m13681983326@163.com); [2767776330@qq.com](mailto:2767776330@qq.com); [camelguo2012@gmail.com](mailto:camelguo2012@gmail.com); [5262579@qq.com](mailto:5262579@qq.com); [ln863@hotmail.com](mailto:ln863@hotmail.com); [quxy75@163.com](mailto:quxy75@163.com); [ywh8708@163.com](mailto:ywh8708@163.com); [Renq275@126.com](mailto:Renq275@126.com);

Corresponding author: Email: [mdcheng18@263.net](mailto:mdcheng18@263.net), Phone:862165690520, Fax: 86-21-65676697

All the blots as follows:


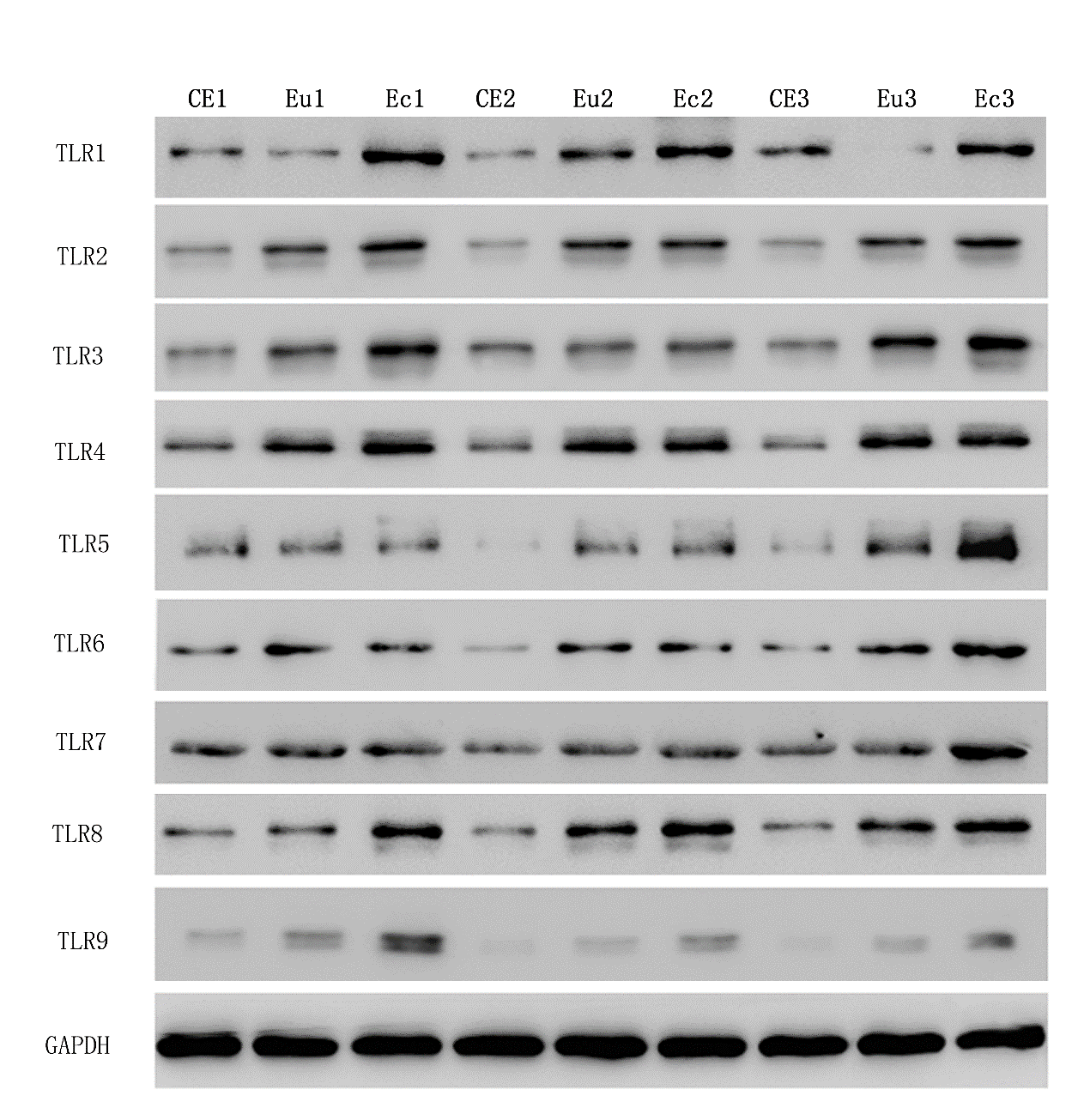


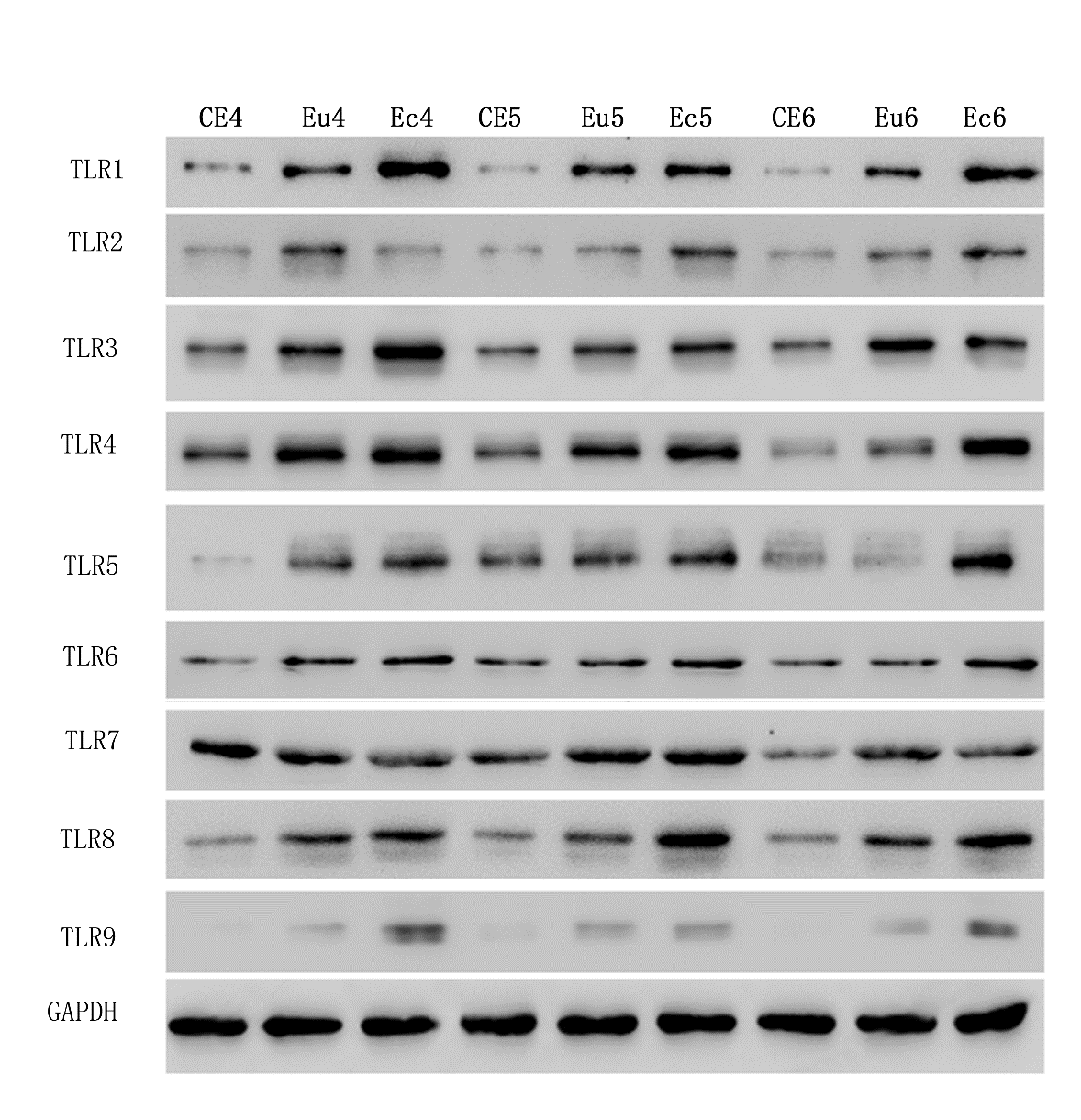


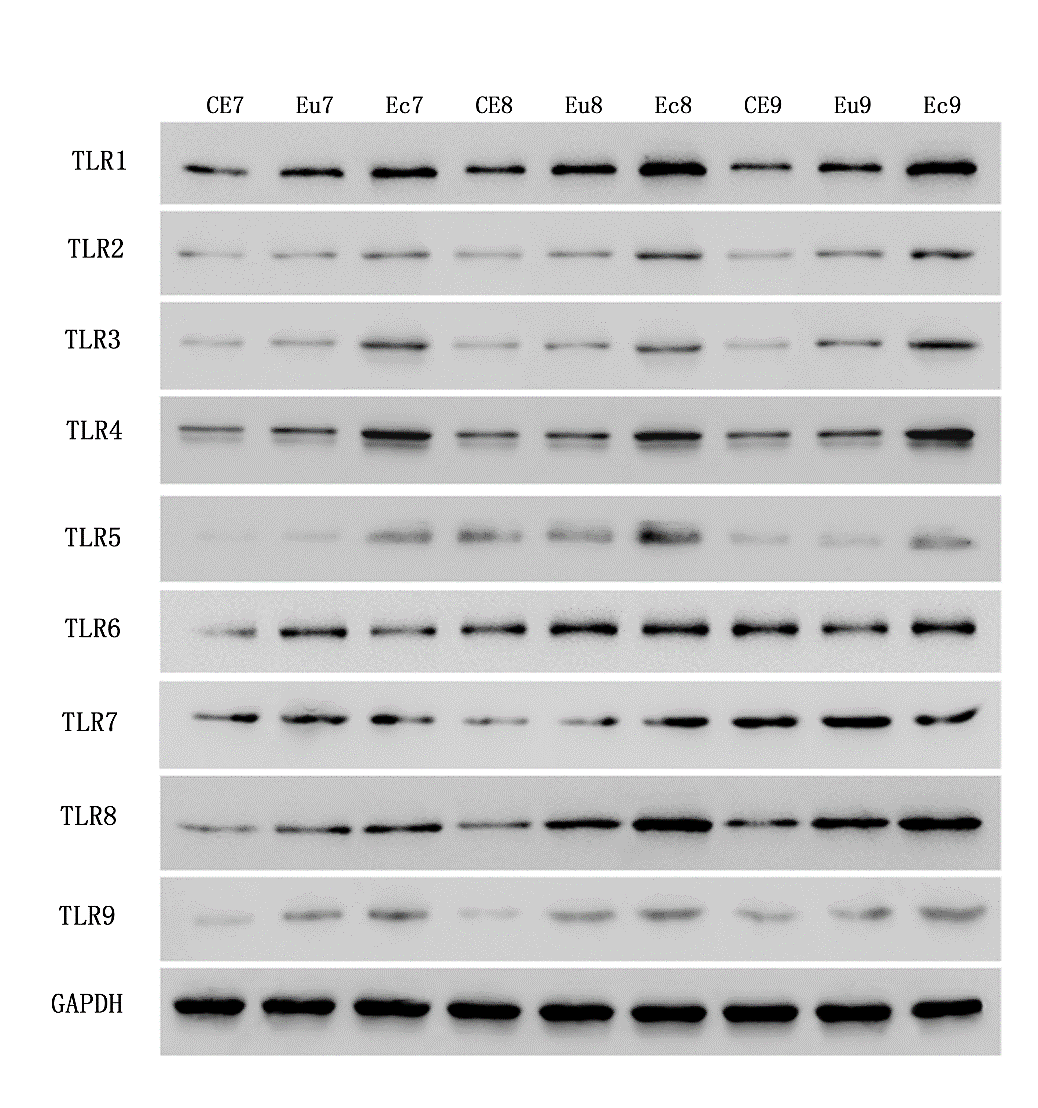


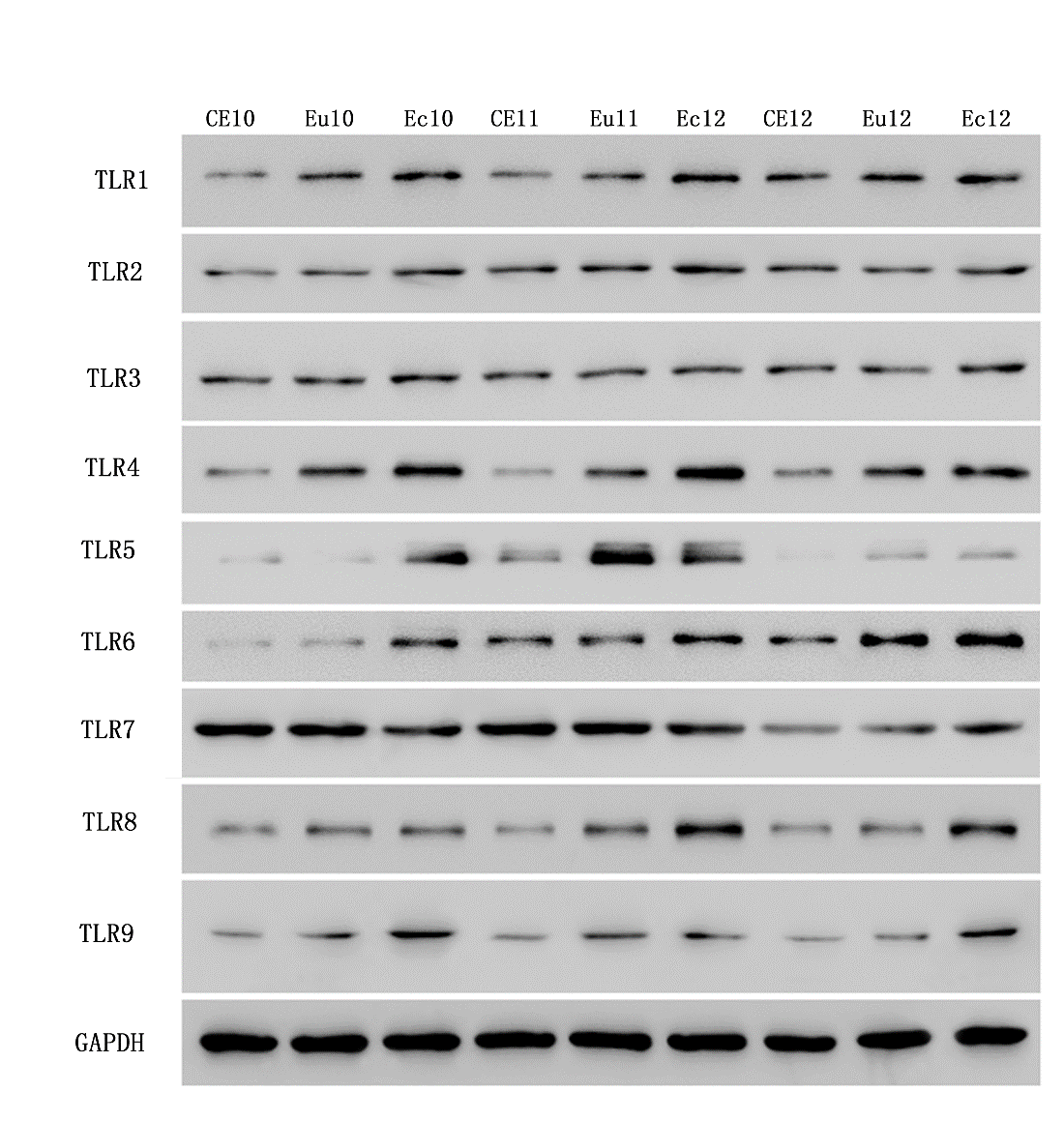


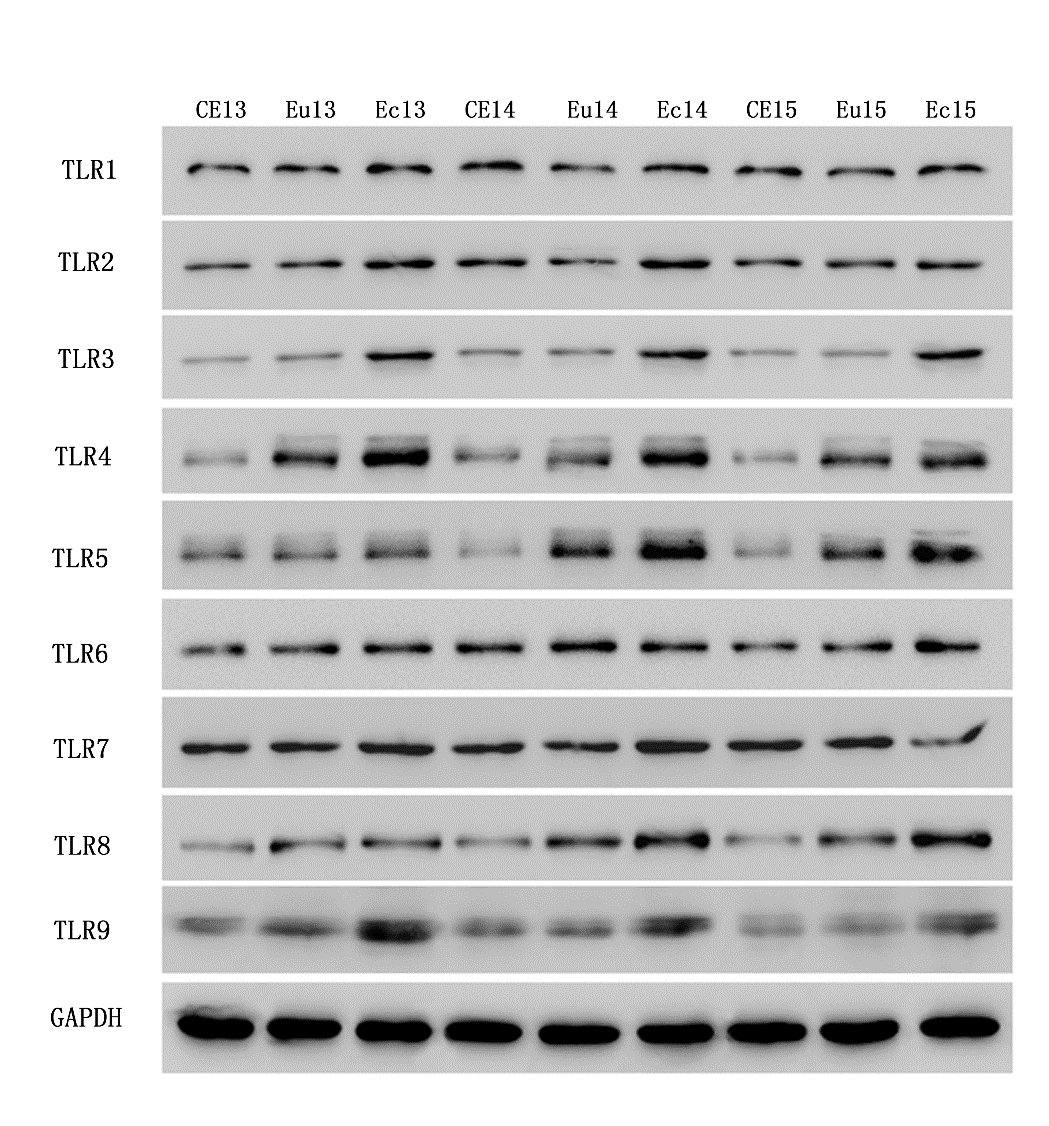


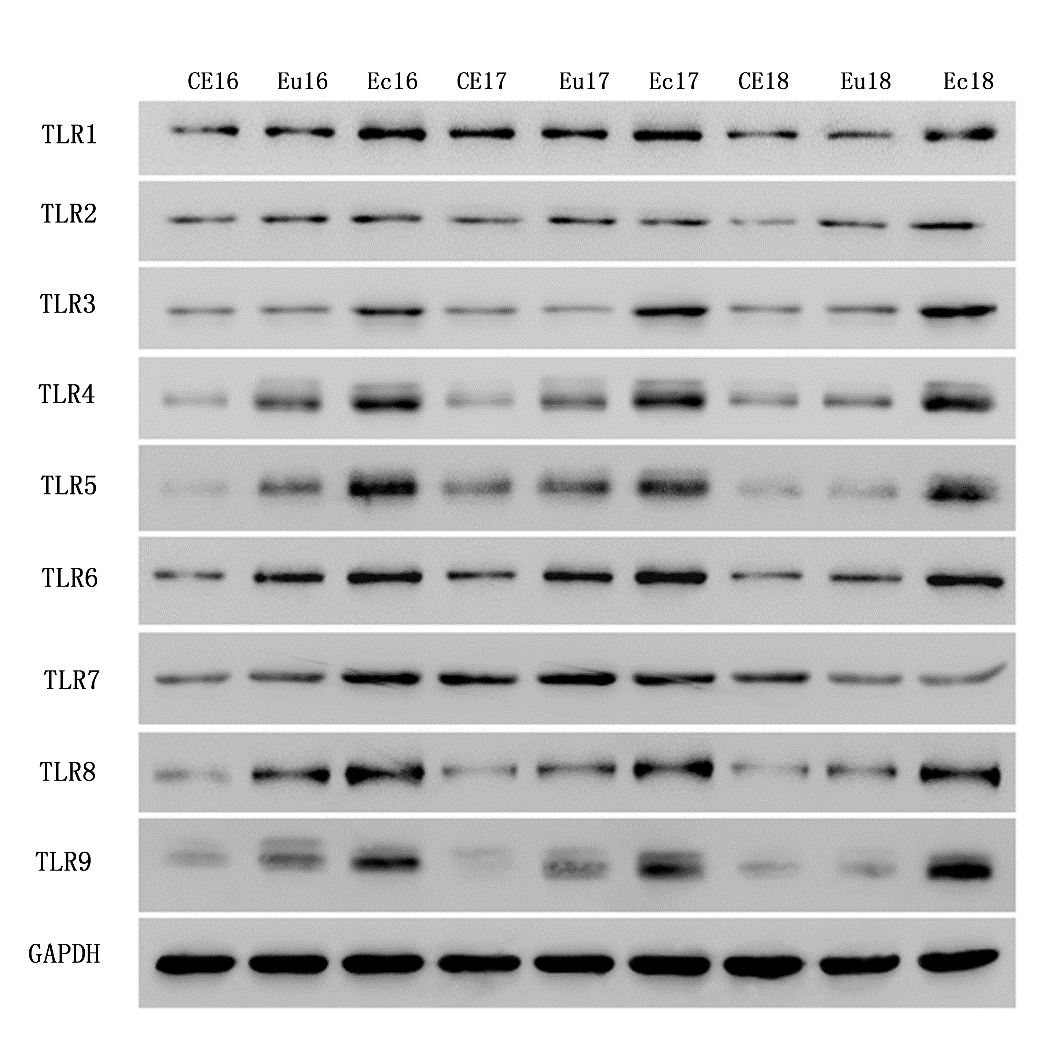


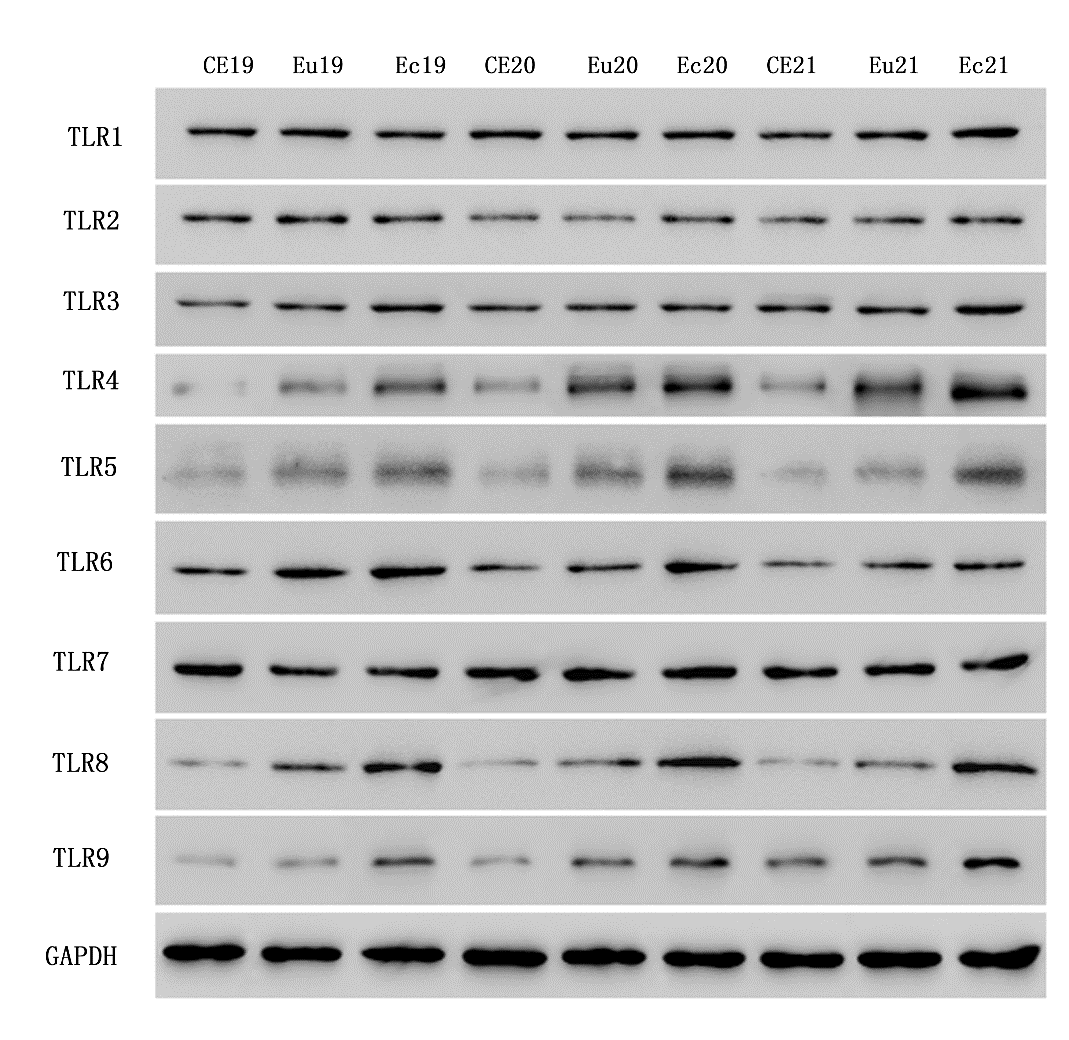


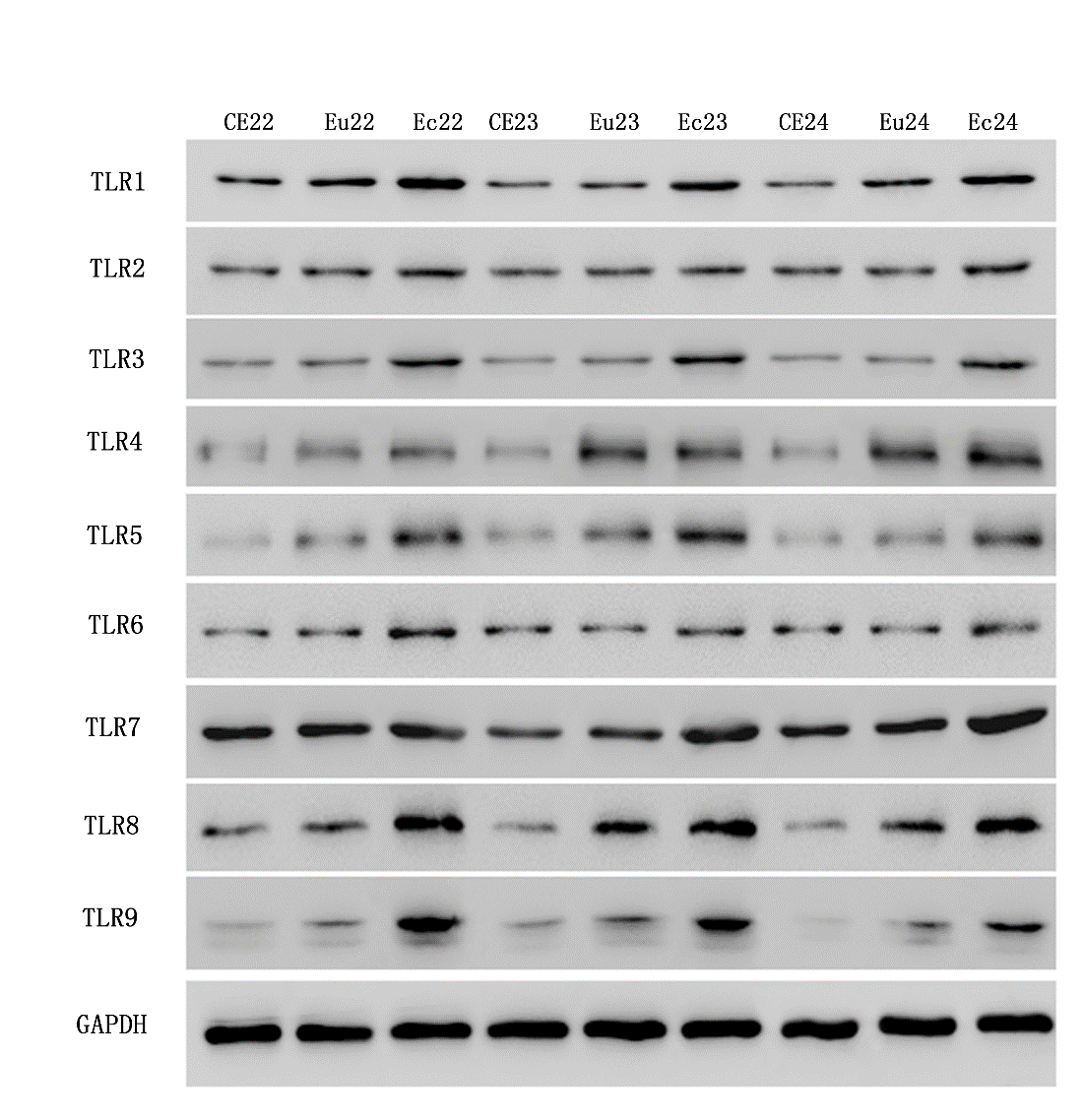


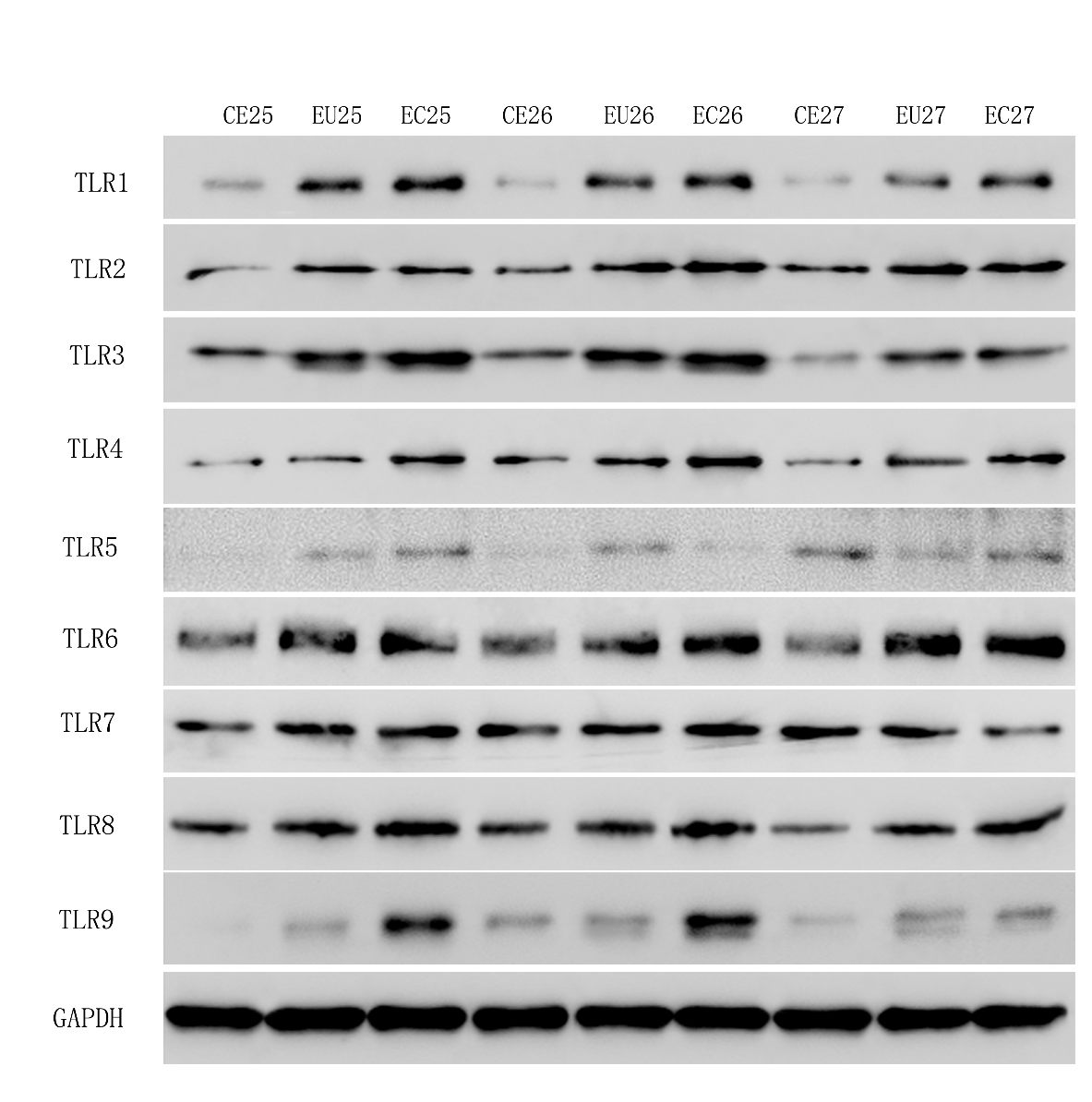


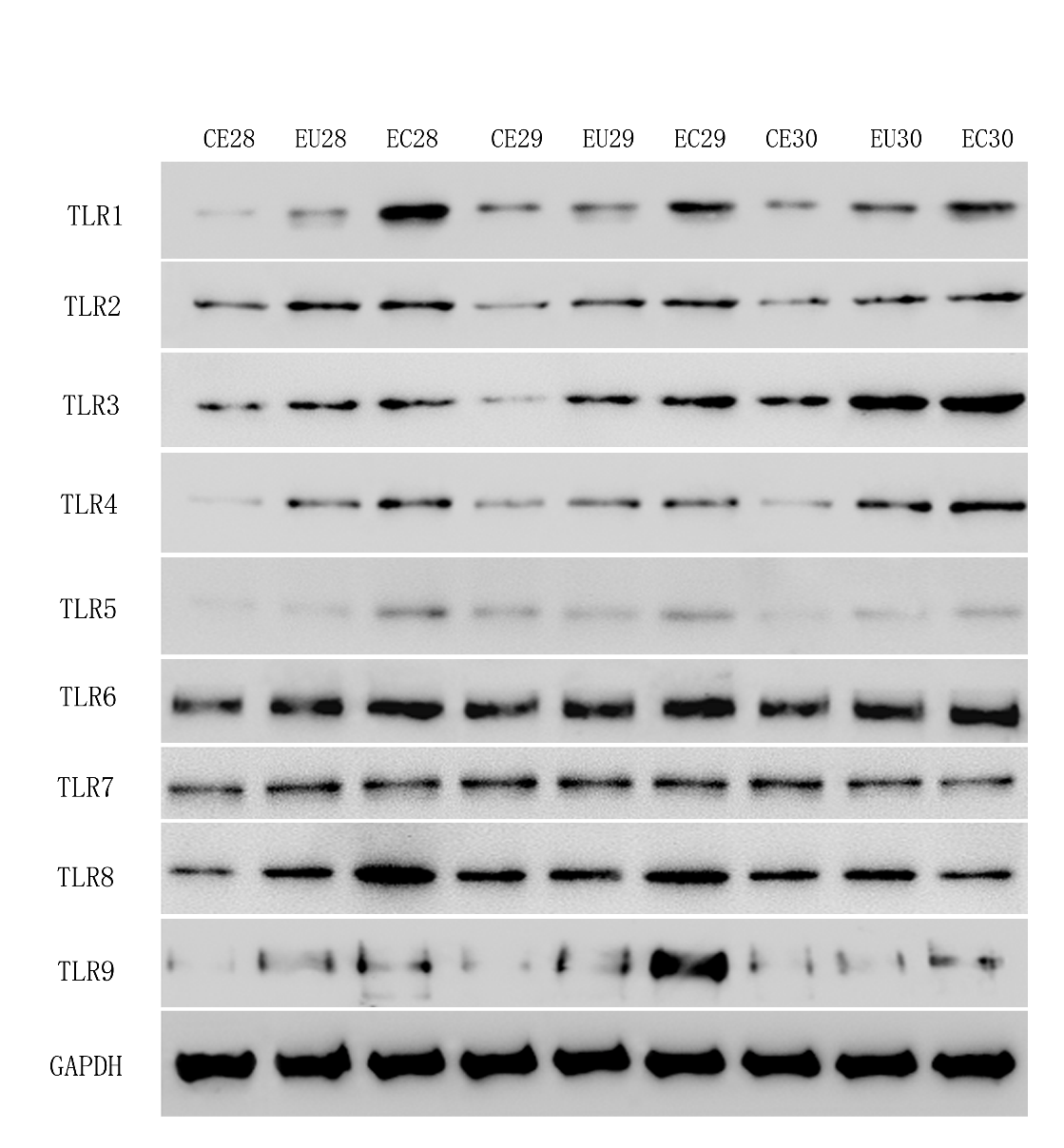

Supplement: Supplementary file 1 — supplementary information [file 41598_2017_7859_MOESM1_ESM.doc]
